# Interventions Intended to Improve the Well‐Being at Work of Nurses Working in Care Settings for Older People—A Systematic Review

**DOI:** 10.1111/opn.70005

**Published:** 2024-12-25

**Authors:** Johanna Wiisak, Arja Suikkala, Helena Leino‐Kilpi, Minna Stolt, Riitta Suhonen, Sanna Koskinen

**Affiliations:** ^1^ Department of Nursing Science University of Turku Turku Finland; ^2^ Diaconia University of Applied Sciences Helsinki Finland; ^3^ Turku University Hospital Turku Finland; ^4^ Satakunta Wellbeing Services County Pori Finland; ^5^ Welfare Services County of Southwest Finland Turku Finland

**Keywords:** intervention, nurse, older people's care, review, well‐being at work

## Abstract

**Introduction:**

Nurses' well‐being at work (WAW) is important for overall health care outcomes. Nurses often navigate complex roles, contending with time constraints, ethical challenges and societal undervaluation, underscoring the necessity of addressing their WAW.

**Methods:**

The aim of this systematic review was to analyse the interventions that potentially improve nurses' WAW in care settings for older people. The ultimate goal is to provide an understanding of this field and advance the development of WAW interventions. We performed a systematic review which was registered in PROSPERO and conducted according to PRISMA guideline. We conducted a comprehensive literature search across five scientific databases and one platform in February 2023.

**Results:**

Out of 5975 records, we included 21 full‐text articles in the review. Interventions were typically complex and focused on (a) nurses' health, (b) nursing care, (c) care facilities and (d) management. Interventions resulted in a range of outcomes on the (a) physical, (b) psychosocial and (c) environmental dimensions of WAW, with most interventions leading to positive outcomes, albeit with instances of negative and neutral results.

**Conclusions:**

Interventions focusing on nurses' health or care facilities can be promising to improve WAW of nurses working in care settings for older people. Interventions aimed at improving the WAW have focused on various aspects. Despite the mainly positive outcomes, some interventions can also compromise nurses' WAW.

**Implications for Practice:**

Strategies and interventions aimed at improving nurses’ WAW are needed in practice as nurses’ WAW is crucial in recruiting to and retaining nurses in care settings for older people. Promoting WAW also contributes to the quality of care for older people and the provision of ethically high‐quality health services.

**Trial Registration:**

The review protocol was registered in the International Prospective Register of Systematic Reviews, PROSPERO (CRD42023399478)

**Summary**

- What does this research add to existing knowledge in gerontology?
  ○ This systematic review provides knowledge of the moderate number of interventions that potentially improve or compromise the well‐being at work (WAW) on the physical, psychosocial and environmental dimensions of nurses working in care settings for older people.
  ○ Interventions focusing on nurses' health or care facilities can be promising to improve nurses' WAW.
- What are the implications of this new knowledge for nursing care for and with older adults?
  ○ The emphasis for the further development, testing and implementation of interventions should be on those interventions that hold potential for enhancing WAW in care settings for older people.
  ○ When planning interventions that focus on changing nursing care practices for older people, nurses' WAW needs to be considered.
- How could the findings be used to influence practice, education, research, and policy?
  ○ Policymakers and managers at various levels can address the improvement of WAW, considering various facets that span from nurses' health to patient care and management practices.
  ○ Research involving nurses' WAW support representatives, including managers, teachers, occupational health professionals and health policymakers, is crucial to efficiently develop and implement interventions for workforce well‐being.
  ○ Nurse educators can more systematically integrate WAW into teaching to cultivate a positive outlook on work among students, emphasising ways to support their well‐being in the workplace.

## 1 Introduction

In care settings for older people, nurses' well‐being at work (WAW) impacts their work productivity; that is, the quality of care provided to patients, patient safety and overall health care outcomes (Organisation for Economic Co‐operation and Development (OECD) 2022; National Academies of Sciences, Engineering, and Medicine (NASEM) 2021). Nurses are the key professionals in promoting health and preventing the worsening of long‐term conditions and particularly maintaining the healthy years of the older population (Rudnicka et al. 2020; Scott 2021), but also in providing care to older people who need long‐term care (OECD 2021). At the time of critical nurse shortage, WAW is crucial in recruiting and retaining nursing staff to respond to the service needs of longevity societies (OECD 2020; Scott 2021). Therefore, nurses' WAW warrants attention.

WAW has various definitions and a consensus seems to lack also in the nursing context. It has been related to all aspects of working life, from workplace health and safety to nurses' subjective judgements, as well as the experience of positive and negative feelings about their work (International Labour Organization (ILO) 2022; OECD 2022). According to previous literature and empirical evidence, WAW is a multidimensional concept with various models proposed (e.g., van Horn et al. 2004; Weziak‐Bialowolska et al. 2020; Patrician et al. 2022). Therefore, in this study, WAW is defined as a positive experience of feeling and being at work, covering physical, psychosocial, ethical and environmental dimensions.

In all care settings, including care services for older people, nurses experience time pressures and work overload, compromising the standards for nursing care due to rationing and prioritisation of scarce resources (Tønnessen, Scott, and Nortvedt 2020). These can subsequently cause physical and mental health problems such as musculoskeletal conditions (OECD 2021) or ethical burdens to nurses (Nikunlaakso et al. 2022), leading to increased absenteeism, turnover and health care costs (OECD 2020). Thus, improving nurses' WAW warrants scrutiny for the benefit of the patients, nurses themselves and wider society.

Nurses' WAW can be improved by all levels of stakeholders, from an individual nurse through the organisation managers to society‐level policymakers with various mechanisms such as self‐care and self‐management programmes, integrating WAW into nurse education, workability management or health policy strategies (NASEM 2021). However, interventions intended to improve nurses' WAW in care settings for older people are overall scarce. Previous reviews of care services for older people have focused on randomised controlled trials (RCT) that promote physical and mental health. (Otto et al. 2021). Physical well‐being interventions were identified as reducing work‐related musculoskeletal disorders (Asuquo, Tighe, and Bradshaw 2021) and workplace physical activity interventions for older employees (Merom et al. 2021). Of psychosocial interventions, reducing burnout (Westermann et al. 2014) and building capacity and resilience (Elliott et al. 2012) were identified. Cognitive‐behavioural and multicomponent interventions were identified as effective approaches to improve nurses' WAW in care services for older people (Westermann et al. 2014; Asuquo, Tighe, and Bradshaw 2021; Otto et al. 2021).

Previous reviews seem to have a limited focus on the physical or psychosocial dimension of WAW or health problems or interventions developed using RCT study designs. Therefore, in this review, we took a broader approach by searching for a spectrum of WAW interventions targeted at nurses working in care settings for older people.

## 2 Materials and Methods

### 2.1 Aim

The aim of this systematic review was to analyse interventions that potentially improve nurses' WAW in care settings for older people. The ultimate goal is to provide an understanding of this field and advance the development of WAW interventions. We set the following questions for this review:

1. What interventions have been developed to improve nurses' WAW?
2. What are the outcomes of WAW interventions?

### 2.2 Design

**FIGURE 1:**
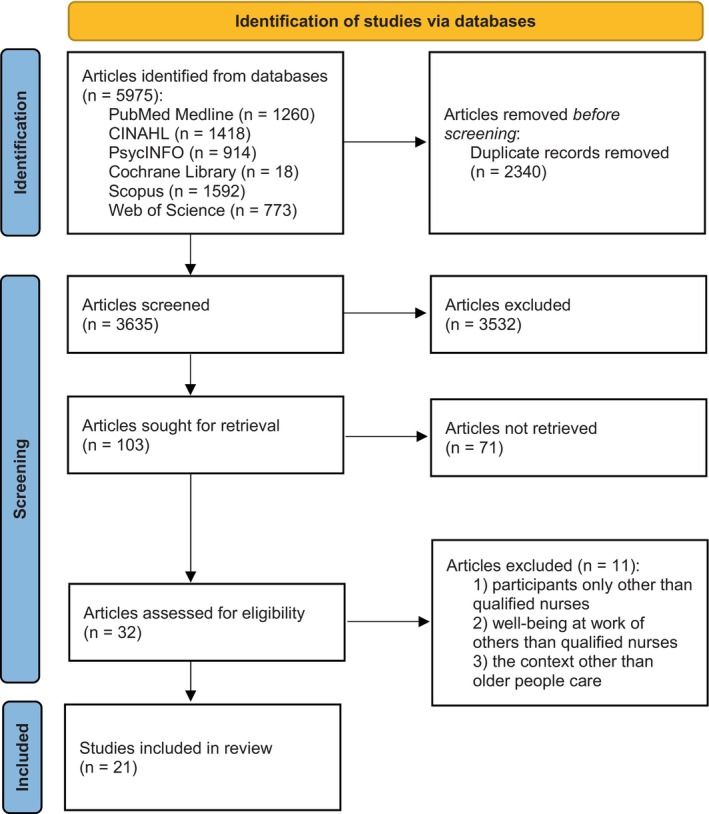
The PRISMA flow diagram, 2020 statement: An updated guideline for reporting systematic reviews (Page et al. 2021).

For this review, we conducted a systematic literature review focusing on interventions improving nurses' WAW in care settings for older people. We followed four stages of The Preferred Reporting Items for Systematic Reviews and Meta‐analyses (PRISMA) guideline (Page et al. 2021; Figure 1).

### 2.3 Search Strategy

In the first identification stage, we conducted a systematic search to identify relevant scientific research articles from the earliest content up to 19 February 2023. We developed search terms and phrases in collaboration with a health and medical science library informatics expert using keywords relating to nurses, WAW and older people's care (Appendix S1). One researcher (JW) undertook the searches in five scientific databases: PubMed Medline, CINAHL (EBSCOhost), PsycINFO, Cochrane Library and Scopus, and in one platform: Web of Science Core Collection. In the second screening stage, two researchers (JW and SK) screened the records on the title and abstract level. In the third eligibility stage, the same researchers retrieved and screened for eligibility against the inclusion and exclusion criteria. In the fourth stage, studies were included in the review and we conducted the backward citation chasing by screening the reference lists of the included articles; however, this did not produce any new articles.

### 2.4 Eligibility Criteria

We included intervention studies (a) that involved qualified nurses (Registered Nurses and licensed practical nurses) as participants among other possible health care professionals, (b) aimed to improve nurses' WAW (covering the physical, psychosocial, ethical or environmental dimension of WAW), (c) that placed in care settings for older people (e.g., nursing homes, home care and long‐term care facilities), (d) that were peer‐reviewed intervention studies with pre–post‐testing, (e) that were written in English and (f) that had the abstract available. Our exclusion criteria were (a) studies that solely involved other health care professionals than qualified nurses such as physicians, psychiatrists or medical students, managers, advanced practice nurses (APN), nurse assistants or nurse aids, (b) studies that focused on well‐being or ill‐being in areas of life other than work life, (c) that focused on hospital care settings and (d) protocols, theoretical articles, literature reviews, books, dissertations, reports, editorials, opinions, discussion papers, study protocols or grey literature.

### 2.5 Quality Appraisal

We conducted quality appraisal using the Joanna Briggs Institute's Critical Appraisal Checklists (Joanna Briggs Institute, JBI 2023) according to the study designs. For 11 studies, we used a randomised controlled trials' (13 items) checklist, and for 10 studies, a quasi‐experimental studies' (9 items) checklist. We ranked the items according to ‘yes’, ‘no’, ‘unclear’ and ‘not applicable’ to describe the appearance of each item in the studies. We scored the studies by assigning one point to the option ‘yes’ and zero points to the other options. We did not exclude any of the articles on a quality basis (Table 1, Appendix S2). Three researchers conducted (JW, AS and SK) quality appraisal, and discrepancies were solved among the research group.

**TABLE 1:** Studies (*n* = 21) included in the review (Registered Nurse, RN; licensed practical nurse, LPN; certified nursing assistant, CAN). Quality appraisal according to Joanna Briggs Institute checklists for quasi‐experimental study designs and randomised controlled trials (JBI 2023).

Authors (year), country	Aim	Study design/methods	Setting sample follow‐up (FU)	Interventions aimed to improve well‐being at work	Quality appraisal score JBI
**Quasi‐experimental**					
Dichter et al. (2017), Germany	To evaluate the effectiveness of the Dementia Care Mapping with regard to caregivers	A pragmatic quasi‐experimental trial with control group	Nursing home units (*n* = 9) in three groups: (a) experience of the method *n* = 3, (b) newly introduced with the method *n* = 3, (c) no experience of the method (control) *n* = 3 RNs and nursing aides *n* = 84 Baseline: (a) *n* = 24, (b) *n* = 28, (c) *n* = 32 Post‐test: (a) *n* = 11, (b) *n* = 16, (c) *n* = 16 18‐month follow‐up: (a) *n* = 7, (b) *n* = 12, (c) *n* = 6	Dementia Care Mapping is a structured, multicomponent and cycling *nursing care* intervention targeting the implementation of person‐centred care in dementia care. Three‐day training course for two caregivers per unit to enhance observation skills; briefing and preparation of the whole caregiver team	6/9^a^
Engst et al. (2005), Canada	To evaluate the risk of injury, job satisfaction and preferred resident handling methods, the frequency and type of resident handling injuries and the cost–benefit of ceiling lift programmes	A quasi‐experimental design with comparison unit	Extended care units of a community hospital *n* = 2 RNs, LPNs, care aides, other Baseline: experimental *n* = 34, control *n* = 34 1‐year follow‐up: experimental *n* = 34, control *n* = 34	*Care facility* intervention of installing care assisting equipment, the ceiling lifts with 1 h training session of their daily use	7/9^a^
Engst et al. (2004), Canada	To evaluate the impact of a scheduled toileting programme on the risk of injury to caregivers and on resident agitation or aggressive behaviours	A quasi‐experimental design with comparison unit	A long‐term care facility units *n* = 2 RNs, care aids, other Baseline: experimental *n* = 61, control *n* = 28 Post‐test: experimental *n* = 43, control *n* = 30	*Nursing care* intervention of scheduled toileting programme, skills training and awareness training on recognition of agitated behaviours	5/9^a^
Ericson‐Lidman and Åhlin (2017), Sweden	To compare assessments of stress of conscience, perceptions of conscience, burnout and social support before and after participation in a participatory action research (PAR) intervention	A follow‐up study without control group	Residential care facilities for older adults; one experimental facility that included four units RNs *n* = 5, nurse assistants *n* = 24 (1‐year follow‐up)	*Nurses' health* intervention focused on psychological issues and used a ‘model of problem processing’ aiming to learn how to deal with troubled conscience. One‐year PAR intervention included 12 sessions including narrating, identifying, prioritising, and brainstorming about situations generating troubled conscience at work. In addition, reviewing knowledge and needs for gaining knowledge for deciding on actions to be undertaken and subsequently evaluating them	6/9^a^
Johnsson, Carlsson, and Lagerström (2002), Sweden	To evaluate the training programme in patient handling and moving skills according to the Stockholm training concept	Pre–post‐test intervention study with control group	Geriatric hospitals, primary care (home care) RNs, state enrolled nurses, occupational therapists, physiotherapists Baseline *n* = 51: traditional groups *n* = 30, quality circles *n* = 21	*Nurses' health* intervention with two models of learning: traditional groups and quality circles, the former involved a course of 4 days and the latter consisted of meetings half a day every second week during 4–6 months, a total of approximately 4 days. Similar content including theoretical and practical parts about patient handling	8/9^a^
Miller et al. (2006), Canada	To examine the impact of installing ceiling lifts in a new long‐term care facility in relation to preferences for patient handling practices and reductions in patient handling injury rates	A quasi‐experimental pre–post‐intervention study with control group	Long‐term care facilities intervention *n* = 1 and comparison *n* = 1 RNs, LPNs, care aides Baseline: intervention *n* = 45, comparison *n* = 29 1‐year follow‐up: experimental *n* = 17, control *n* = 15	*Care facility* intervention of portable ceiling lifts as patient handling equipment and 1‐hour training of their daily use	5/9^a^
Nelson et al. (2006), USA	To evaluate the impact of the multifaceted programme integrating evidence‐based practice, technology and safety improvement on injury rate, lost and modified workdays, job satisfaction, self‐reported unsafe patient handling acts, level of support for programme, staff and patient acceptance, programme effectiveness, costs and return on investment	A pre−post‐intervention study without a control group	High‐risk units *n* = 23: nursing home units *n* = 19 and spinal cord injury units *n* = 4 RNs, LPNs, nursing aides, student nurses, health care technicians assigned to perform patient care, nurse managers performing direct patient care Baseline *n* = 209 9‐month follow‐up *n* = 210	*Nurses' health* intervention of multifaceted programme with six programme elements about patient handling: (1) ergonomic assessment protocol, (2) patient handling assessment criteria and decision algorithms, (3) peer safety leaders, known as a Back Injury Resource Nurses (BIRNS), (4) patient handling equipment based on needs identified in the ergonomic assessment, (5) after action review (AAR) process and (6) no lift policy	4/9^a^
Petterson, Lagerström, and Toomingas (2006), Sweden	To evaluate the impact of an empowering education intervention programme at individual, worksite and organisational level for nursing staff in older people services	A prospective intervention study without control group	Older people care units *n* = 12: home care *n* = 3 and nursing homes *n* = 9 RNs, auxiliary or assistant nurses, other *n* = 200 (baseline and 18‐month follow‐up): home care staff *n* = 73 and nursing home staff *n* = 127	Educational 18‐month *nurses' health* intervention programme was carried out in three steps: (a) a competence programme for selected group of nurses, (b) worksite competence circles for all staff and (c) local worksite projects for separate worksites and the organisation	6/9^a^
Sarabia‐Cobo et al. (2017), Spain	To test the impact of theoretically based training on the different components of emotional intelligence and coping styles	An intervention study with pre‐ and post‐tests and follow‐up without control group	Nursing homes RNs, CNAs *n* = 92 (baseline, post‐test, 1‐year follow‐up)	*Nurses' health* intervention of the psychological training had four 4‐h sessions over a 4‐week period about (1) perception, appraisal and expression of emotion; (2) emotional facilitation of thinking; (3) understanding and analysing emotions; and (4) reflective regulation of emotion	7/9^a^
Zwakhalen et al. (2018), the Netherlands	To study the effects of working in a new type of dementia care facility (i.e., small‐scale living facilities) on burnout symptoms and job characteristics (job autonomy, social support, physical demands and workload)	A quasi‐experimental, longitudinal study with control group	Small‐scale living facilities *n* = 28 and regular psychogeriatric wards in nursing homes *n* = 21 RNs, CNAs, nursing assistants, basic nursing aids Baseline: experimental *n* = 114, control *n* = 191 6‐month follow‐up: experimental *n* = 72, control *n* = 109 12‐month follow‐up: experimental *n* = 69, control *n* = 87	Small‐scale *care facility*: 1. Maximum of eight residents per house or unit 2. Staff, residents and their family form a household together and activities are centred around the daily life and household 3. Staff perform integrated tasks 4. Residents are cared for by a small, fixed team of nursing staff 5. Daily life is organised completely or to a large extent by residents, their family caregivers and nursing staff 6. The archetypal home is a physical setting that resembles a homelike environment	6/9^a^
**Randomised controlled trials**					
Berendonk et al. (2019), Germany	To test the feasibility of a nursing intervention (DEMIAN) in routine care and its effects on job satisfaction, motivation and work strain	A cluster‐randomised trial with control group	Long‐term care facilities Experimental *n* = 10, control *n* = 10 RNs, care providers Baseline: experimental *n* = 84, control *n* = 96 8‐week follow‐up: experimental *n* = 71, control *n* = 87	*Nursing care* intervention including 2 days of training within 2 weeks and a 6‐week implementation phase during which trained Registered Nurses and care providers provided emotion‐focused mini‐interventions for participating residents	6/13^b^
Berg, Welander Hansson, and Hallberg (1994), Sweden	To study creativity and innovative climate, tedium and burnout during 1‐year of systematic clinic supervision combined with the implementation of individualised care for severely demented patients, as compared with a similar control ward	Pre–post‐test intervention study with control group	Psychogeriatric clinic Experimental *n* = 1, control *n* = 1 Baseline *n* = 39 RNs: experimental *n* = 4, control *n* = 4 Licensed practical nurses: experimental *n* = 13, control *n* = 12 Other: experimental *n* = 2, control = 4 6‐month follow‐up *n* = 32 12‐month follow‐up *n* = 31	Individually planned and documented *nursing care* combined with regular systematic clinical supervision. Two days course for nurses and carers followed by another 2 days during the year of the study	3/13^b^
Buruck et al. (2016), Germany	To evaluate the impact of a standardised emotion regulation training (affect regulation training) to improve emotion regulation skills and well‐being	A 2 × 3 factor repeated measures design (ART/control group vs. pre‐/post‐/follow‐up assessment)	Nursing homes *n* = 14 Nurses and carers *n* = 96 Baseline: experimental *n* = 38, control *n* = 44 Post‐test: experimental *n* = 26, control *n* = 44 6‐month follow‐up *n* = 16, control *n* = 12	*Nurses' health* intervention includes cognitive‐behavioural and stress relaxation techniques and adds mindfulness‐based strategies to improve stress regulation and emotion regulation with seven emotion regulation skills	3/13^b^
Davison et al. (2007), Australia	To evaluate the impact of a training programme in managing dementia‐related challenging behaviours	Pre–post‐test intervention study with control group	High‐level care facilities *n* = 2 and low‐level care facilities *n* = 2 Baseline: Nurses and nursing assistants *n* = 90 Training and peer support *n* = 29, training *n* = 35, control *n* = 26	*Nursing care* intervention included five peer support sessions and eight‐session dementia training on challenging behaviours	4/13^b^
Faghri et al. (2017), USA	To evaluate if self‐efficacy and financial incentives mediate the effect of health behaviour on weight loss	A randomised cluster design with control group	Long‐term care facilities, nursing homes *n* = 4 Nurses and other *n* = 99 (baseline, 1, 16 and 28 weeks follow‐ups)	*Nurses' health* intervention of 16‐week workplace‐based weight loss programme for employees included personalised weight‐loss consultation based on reported physical activity habits and dietary preferences. Each participant received an action plan, which included diet and activity tracker encouraging them and providing information on safe weight loss, goal setting, healthy eating and increasing physical activity	6/13^b^
Hurtado et al. (2016), USA	To examine effects on cigarette consumption of a work–family supportive organisational intervention	A group‐randomised controlled trial with control group	Nursing homes Experimental *n* = 15, control *n* = 15 RNs, LPNs, CNAs *n* = 1524 Baseline: experimental *n* = 725, control *n* = 687 6‐month follow‐up: experimental *n* = 626, control *n* = 678	*Nurses' health* STAR (Support; Transform; Achieve; Results) intervention was based on social and organisational changes aimed at enhancing employees' control over work hours and increase supervisory support for employees' work–family concerns. The intervention was delivered over 4 months per nursing home. Delivery occurred in three stages: (1) preparing for change, (2) setting the change in motion and (3) sustaining the change	6/13^b^
Jeon et al. (2015), Australia	To determine the effectiveness of an aged care‐specific leadership and management programme [the Clinical Leadership in Aged Care (CLiAC)]	A double‐blind cluster‐randomised controlled trial with control group	Residential care *n* = 12, community care *n* = 12 RNs, care supervisors, personal care assistant/assistant in nursing, other Baseline: experimental *n* = 202, control *n* = 301 9‐month follow‐up: experimental *n* = 229, control *n* = 315 18‐month follow‐up: experimental *n* = 240, control *n* = 342	*Managerial* 12‐month CLiAC a structured education and support programme for aged care middle managers to promote safe, high‐quality person‐centred and evidence‐based care by assisting middle managers to develop effective team relationships and person‐/client‐centred leadership strategies that enable them to deal with the day‐to‐day realities of care service utilising action learning techniques, 360° feedback, case scenarios, one‐on‐one interactions with a programme facilitator and individual practice improvement	12/13^b^
Kloos, Drossaert, and Bohlmeijer (2019), the Netherlands	To test the effectiveness and acceptability of an online multicomponent positive psychology intervention	A cluster‐randomised controlled design with control group	Nursing homes Experimental *n* = 2, control *n* = 2 RNs, LPNs, nurse assistants, students *n* = 159 Baseline: experimental *n* = 79, control *n* = 77 12‐week follow‐up: experimental *n* = 63, control *n* = 36	Online gamified multicomponent *Nurses' health* positive psychology intervention lasting 8–12 weeks. Eight modules covering six key topics of well‐being: (1) positive emotions; (2) discovering and using strengths; (3) optimism; (4) self‐compassion; (5) resilience and (6) positive relations. Each module consists of psycho‐education and approximately five evidence‐based positive psychology exercises that can be completed multiple times	6/13^b^
Torsney (2011), USA	To investigate the impact of including certified nursing assistants and licensed practical nurses in interdisciplinary team meetings on stress and coping	A pre‐ and post‐test control group pilot study with control group	Long‐term care units *n* = 10 LPNs, CNAs *n* = 46 Experimental LPN *n* = 11, CNA *n* = 12 Control LPN *n* = 11, CNA *n* = 12	*Nurses' health* intervention of interdisciplinary team meetings	4/13^b^
Tveito and Eriksen (2009), Norway	To assess if an Integrated Health Programme would reduce sick leave and subjective health complaints and increase coping.	A randomised controlled pilot study with control group	Nursing home *n* = 1 Nursing auxiliaries, assistants without formal education, other Baseline: experimental *n* = 19, control *n* = 21 Post‐test: experimental *n* = 12, control *n* = 17	*Nurses' health* intervention consisted of three main parts: (1) physical exercise, (2) health information/stress management training and (3) a practical examination of the workplace	11/13^b^
Zimmerman et al. (2010), USA	To evaluate the components of the Foundations of Dementia Care programme	A nested cohort group‐randomised trial	Nursing homes *n* = 9 and residential care/assisted living settings *n* = 7 RNs, LPNs, others Experimental *n* = 213, control *n* = 278 Supervisors Experimental *n* = 78, control *n* = 93 (Pre‐ and post‐tests, 3‐month follow‐up)	A 6‐week *nursing care* training with three modules taught through six sessions. Module 1) learning to lead: leading the team (attended only by supervisors); classroom to practice (attended only by supervisors); building a vision (attended by supervisors and direct care staff). Module 2) about dementia: improving communication (attended by supervisors and direct care staff). Module 3) reducing pain: Pain awareness; pain practice (attended by supervisors and direct care staff)	9/13^b^

*Note:* Quality appraisal: ^a^JBI checklist for quasi‐experimental study designs nine items; ^b^JBI checklist for randomised controlled trials thirteen items.

### 2.6 Data Management

We used Zotero software (Roy Rosenzweig Center for History and New Media 2016) to store and handle the articles.

### 2.7 Data Extraction and Analysis

We conducted narrative data analysis complemented with summative tables as the included studies regarding interventions and outcomes were heterogeneous (Graneheim and Lundman 2004). First, we extracted the data of the selected articles into a table format worksheet that we developed (JW and SK). The extracted data included author(s), year, country, aim(s), study design, setting, sample, methods, data collection, data analysis, descriptions of the interventions and identification of their focus (Table 1). Second, we categorised information about the outcomes of the interventions according to the physical, psychosocial and environmental dimensions of WAW. Third, we analysed the statistically significant effects of the interventions on the outcomes according to + positive effect, = no effect, − negative effect on each dimension of WAW (Table 2). One researcher (JW) extracted and analysed the data independently, while another (SK) checked these data. We solved disagreements by discussion, and those not resolved were referred to the research group. Without making interpretations, we used the original expressions used by authors in their articles.

**TABLE 2:** Outcomes (statistically significant) of the interventions according to the dimensions of well‐being at work (WAW). Outcome effect positive (+), negative (−), no effect (0).

Intervention focus	WAW dimension	Effect	Outcomes
Nurses' health	Physical	+	Less neck complaints (Tveito and Eriksen 2009)^b^ Reduction in cigarette consumption (Hurtado et al. 2016)^b^ Improvements in muscle pain (Tveito and Eriksen 2009)^b^ Decrease in musculoskeletal injuries (Nelson et al. 2006)^a^ Improvements in physical fitness (Tveito and Eriksen 2009)^b^ Weight loss (Faghri et al. 2017)^b^ Physical exertion decreased (Johnsson, Carlsson, and Lagerström 2002)^a^ Less discomfort in patient transfers (Johnsson, Carlsson, and Lagerström 2002)^a^ Improvements in work technique (Johnsson, Carlsson, and Lagerström 2002)^a^
		0	No decrease in musculoskeletal problems^b^ (Johnsson, Carlsson, and Lagerström 2002)^a^
	Psychosocial	+	Increased job satisfaction (Nelson et al. 2006, ^a^; Kloos, Drossaert, and Bohlmeijer 2019, ^b^; Tveito and Eriksen 2009, ^b^) Increase in coping styles (Sarabia‐Cobo et al. 2017)^a^ Fosters emotion regulation skills (Buruck et al. 2016)^b^ Increase in emotional intelligence (Sarabia‐Cobo et al. 2017)^a^ Improvements in stress management (Tveito and Eriksen 2009)^b^
		0	No impact on burnout (Ericson‐Lidman and Åhlin 2017)^a^ Stress (Ericson‐Lidman and Åhlin 2017)^a^ Work engagement (Kloos, Drossaert, and Bohlmeijer 2019)^b^ No decrease on job strain (Johnsson, Carlsson, and Lagerström 2002)^a^
	Environmental	+	Modified duty days taken by injury (Nelson et al. 2006)^a^ Decrease in the number of ‘unsafe’ patient handling practices performed daily (Nelson et al. 2006)^a^ Greater task‐centred coping for lower status workers (Torsney 2011)^b^ Improvements in maintenance of work situation (Tveito and Eriksen 2009)^b^ Improved work conditions (Petterson, Lagerström, and Toomingas 2006)^a^
		0	Staff resources (Petterson, Lagerström, and Toomingas 2006)^a^ No effects on sick leaves (Tveito and Eriksen 2009)^b^ No reduction on the number of lost workdays (Nelson et al. 2006)^a^
Nursing care	Physical	+	Reduction in perceived risk of injury (Engst et al. 2004)^a^
	Psychosocial	+	Decreased job dissatisfaction (Berendonk et al. 2019)^b^ Tedium decreased (Berg, Welander Hansson, and Hallberg 1994)^b^ Burnout decreased (Berg, Welander Hansson, and Hallberg 1994)^b^ Improved attitudes when caring residents with challenging behaviours (Davison et al. 2007)^b^
		0	No impact on burnout (Davison et al. 2007)^b^ Stress (Dichter et al. 2017)^a^
		−	Increase in work stress (Zimmerman et al. 2010)^b^ Burnout increased (Dichter et al. 2017)^a^ Positive attitudes toward dementia decreased (Dichter et al. 2017)^a^ Job satisfaction demonstrated a negative development (Dichter et al. 2017)^a^ Less support from supervisors (Zimmerman et al. 2010)^b^
	Environmental	+	Decreased time pressure at work (Berendonk et al. 2019)^b^
		−	Increased workload (Engst et al. 2004)^a^
Care facilities	Physical	+	Reduction in perceived risk of injury (Miller et al. 2006)^a^
		0	Not reducing the perceived risk of injury when repositioning residents (Engst et al. 2005)^a^
	Psychosocial	+	Increased job satisfaction (Engst et al. 2005)^a^ More job autonomy (Zwakhalen et al. 2018)^a^
		0	Social support (Zwakhalen et al. 2018)^a^ No impact on burnout (Zwakhalen et al. 2018)^a^
	Environmental	+	Lower workload (Zwakhalen et al. 2018)^a^ Fever physical demands (Zwakhalen et al. 2018)^a^
Management	Psychosocial	+	Perceived management support (Jeon et al. 2015)^b^
	Environmental	0	No reduction in staff turnover (Jeon et al. 2015)^b^

^a^ Quasi‐experimental study design.

^b^ Randomised controlled trial design.

## 3 Results

We discovered 5975 database records, of which we removed 2340 as duplicates. Then, we screened a total of 3635 articles by title and abstract excluding 3532 articles. We retrieved a total of 103 full‐text reports, of which 82 reports were excluded according to full texts. Finally, we included 21 studies in the review (Figure 1).

The overall quality of the studies was good in quasi‐experimental studies (mean scores 6/9; range 4–8/9) and moderate in RCTs (mean scores 6.4/13; range 3–12/13) (Table 1). None of the studies received full scores and some scored very low. According to item‐level scores, the most common weaknesses in quasi‐experimental studies were in the use of appropriate statistical analysis and blinding in RCTs (Appendix S2).

### 3.1 Characteristics of the Studies

The studies (*n* = 21) were published between 1994 and 2019; nearly all (*n* = 20) were published in the 2000s. The studies were carried out in the United States (*n* = 5), Sweden (*n* = 4), Canada (*n* = 3), Germany (*n* = 3), Australia (*n* = 2), the Netherlands (*n* = 2), Norway (*n* = 1) and Spain (*n* = 1) (Table 1).

The study designs were RCTs (*n* = 11) and quasi‐experimental (*n* = 10). There was a control group in 17 studies. A post‐test was carried out in three studies immediately after the intervention ended. Five studies had immediate post‐tests with one follow‐up measurement (range: 3 months to 1 year). Most commonly, studies (*n* = 9) included a pre‐test with one follow‐up measurement (range: 8 weeks to 18 months). Four studies had pre‐tests with two follow‐up measurements (range: 1 week to 18 months) (Table 1).

As for the contexts in which the interventions were carried out, different names were used for corresponding older people's care services in each country. The interventions were implemented most often in nursing homes (*n* = 10) or long‐term care facilities (*n* = 6) (Table 1).

The participants were qualified nurses (*n* = 21), either Registered Nurses (*n* = 16) or licensed practical nurses (*n* = 12). However, all the studies also included nurse aides or nursing assistants (*n* = 14), other health care staff (*n* = 11), nurse managers (Jeon et al. 2015; Nelson et al. 2006; Zimmerman et al. 2010) or nursing students (Kloos, Drossaert, and Bohlmeijer 2019; Nelson et al. 2006). The number of participants who participated in all pre‐ and post‐tests and follow‐ups in experimental groups ranged between 7 and 626, and in control groups, between 6 and 678 (Table 1).

### 3.2 Interventions Developed to Improve the Nurses' WAW

The majority (*n* = 20) of the interventions included more than one component. One intervention was implemented in existing care facilities without other components (Zwakhalen et al. 2018). Nearly, all interventions included traditional classroom education (*n* = 19). In addition, one intervention included gamified training (Kloos, Drossaert, and Bohlmeijer 2019), and one, computer‐based training (Hurtado et al. 2016). Several interventions included active learning strategies, either as group or individual activities or exercises. Consultation was used in one intervention (Faghri et al. 2017). In two interventions, care equipment was installed (Engst et al. 2004; Miller et al. 2006) (Table 1).

The interventions developed to improve the nurses' WAW focused on (a) nurses' health (*n* = 11), (b) nursing care (*n* = 6), (c) care facilities (Engst et al. 2005; Miller et al. 2006; Zwakhalen et al. 2018) and (d) management (Jeon et al. 2015) (Tables 1 and 2). Nurses' health covered interventions aimed to improve mental (Torsney 2011; Buruck et al. 2016; Ericson‐Lidman and Åhlin 2017; Berendonk et al. 2019; Sarabia‐Cobo et al. 2017; Kloos, Drossaert, and Bohlmeijer 2019) or physical health (Johnsson, Carlsson, and Lagerström 2002; Engst et al. 2005; Miller et al. 2006; Nelson et al. 2006; Tveito and Eriksen 2009; Hurtado et al. 2016; Faghri et al. 2017) such as emotion regulation, weight loss (Faghri et al. 2017) or decreased cigarette consumption (Hurtado et al. 2016). Interventions that focused on nursing care were about introducing new practices for dementia care (Engst et al. 2004; Davison et al. 2007; Zimmerman et al. 2010; Dichter et al. 2017) or individualised care (Berg, Welander Hansson, and Hallberg 1994) that aimed to decrease nurses' burnout, job strain or workload. Interventions focusing on care facilities by installing ceiling lifts (Engst et al. 2005; Miller et al. 2006) or small‐scale living facilities (Zwakhalen et al. 2018) as an intervention aimed to prevent injuries or decrease negative symptoms such as burnout. Interventions in the area of management focused on management development programmes for middle‐level managers (Jeon et al. 2015) that aimed to decrease work stressors and staff turnover rates (Table 1).

### 3.3 Outcomes of the Interventions Developed for Nurses' WAW

The outcomes of the nurses' health, nursing care, care facilities and management‐focused interventions for nurses' WAW varied in the studies. Most (*n* = 18) of the interventions showed some positive outcomes, while three showed negative ones (Engst et al. 2004; Zimmerman et al. 2010; Dichter et al. 2017) and nearly half showed also outcomes with no effect (*n* = 10) (Table 1).

Many outcomes of the (a) physical, (b) psychosocial and (c) environmental dimensions of WAW were studied with the emphasis being on the psychosocial dimension. Both quasi‐experimental and RCT studies showed some positive outcomes in three dimensions of WAW. Intervention studies with low or high quality all showed some positive outcomes, except for one high‐quality RCT that showed only negative outcomes (Zimmerman et al. 2010) (Table 1). Interventions that focused on nurses' health, nursing care or care facilities showed positive outcomes in all three dimensions of WAW. However, interventions that focused on nursing care also showed some negative outcomes in the psychosocial and environmental dimensions of WAW. In addition, interventions that focused on nurses' health, nursing care, care facilities or management all showed no effect on some of the three dimensions of WAW (Table 2).

*In the physical dimension* of WAW, positive outcomes of the interventions that focused on nurses' health, nursing care or care facilities decreased physical exertion (Johnsson, Carlsson, and Lagerström 2002), discomfort in patient transfers (Engst et al. 2005; Johnsson, Carlsson, and Lagerström 2002) or cigarette consumption (Hurtado et al. 2016) as well as improved weight loss (Faghri et al. 2017), physical fitness (Tveito and Eriksen 2009) or work techniques (Johnsson, Carlsson, and Lagerström 2002). Differences in the results were reported regarding musculoskeletal problems as interventions focused on nurses' health decreased (Nelson et al. 2006) or nursing care interventions had no effects on them (Johnsson, Carlsson, and Lagerström 2002). Contradicting results were reported about the risk of injury, which was either reduced by nursing care interventions (Engst et al. 2004; Miller et al. 2006) or not reduced by interventions focused on care facilities (Engst et al. 2005).

*In the psychosocial dimension* of WAW, interventions that focused on nurses' health, nursing care or care facilities decreased tedium (Berg, Welander Hansson, and Hallberg 1994) as well as improved coping styles (Sarabia‐Cobo et al. 2017), emotion regulation skills (Buruck et al. 2016), emotional intelligence (Sarabia‐Cobo et al. 2017) or job autonomy (Zwakhalen et al. 2018). However, there were differences in the results concerning burnout (Berg, Welander Hansson, and Hallberg 1994; Davison et al. 2007; Dichter et al. 2017; Ericson‐Lidman and Åhlin 2017; Zwakhalen et al. 2018), stress (Dichter et al. 2017; Ericson‐Lidman and Åhlin 2017; Tveito and Eriksen 2009; Zimmerman et al. 2010) and social support (Jeon et al. 2015; Zimmerman et al. 2010; Zwakhalen et al. 2018), which were improved by nurses' health and management‐focused interventions and decreased by nursing care interventions, or nurses' health, nursing care and care facilities‐focused interventions had no effect on these. Contradicting results were reported about job satisfaction (Berendonk et al. 2019; Dichter et al. 2017; Kloos, Drossaert, and Bohlmeijer 2019; Nelson et al. 2006) or positive attitudes that either improved when caring for residents with challenging behaviours by nurses' health, nursing care and care facilities‐focused interventions (Davison et al. 2007) or decreased towards dementia (Dichter et al. 2017) by nursing care interventions. Nurses' health and nursing care‐focused interventions had no effect on job strain (Johnsson, Carlsson, and Lagerström 2002) or work engagement (Kloos, Drossaert, and Bohlmeijer 2019).

*In the environmental dimension*, interventions that focused on nurses' health, nursing care or care facilities decreased adjusted duty days caused by injury (Nelson et al. 2006), physical demands (Zwakhalen et al. 2018), time pressure at work (Berendonk et al. 2019) and unsafe care practices (Nelson et al. 2006) and improved work conditions (Petterson, Lagerström, and Toomingas 2006), the maintenance of the work situation (Tveito and Eriksen 2009) and task‐centred coping (Torsney 2011). Results in the studies differentiated concerning workload, which either decreased by intervention focused on care facilities (Zwakhalen et al. 2018) or increased by nursing care intervention (Engst et al. 2004). Nurses' health or management‐focused interventions had no effects on staff resources (Petterson, Lagerström, and Toomingas 2006), the number of lost workdays (Nelson et al. 2006), sick leaves (Tveito and Eriksen 2009) and turnover (Jeon et al. 2015).

## 4 Discussion

In this review, we analysed interventions that aimed to improve nurses' WAW in care settings for older people. This knowledge is valuable, as more RCTs in the field of nurses' WAW are needed at all levels: individual, organisation and health system (NASEM 2021). In addition, improving WAW through interventions is critical for recruiting and retaining nurses as well as orienting their careers not only to all care settings, but also to care settings for older people where there is a severe shortage of qualified nurses (Nikunlaakso et al. 2022).

The moderate number of intervention studies that we uncovered in this review underscores the imperative for additional intervention testing, as it is pivotal in fortifying the evidence base, particularly in the light of the recognised relevance of WAW to the nursing profession and provision of high‐quality care for older people (OECD 2022).

Some studies showed poor quality which carries implications for the future utilisation of the review findings. Corresponding weakness was discovered in a previous review on well‐being interventions (Melnyk et al. 2020). In the current review, methodological failings also have to do with short follow‐up periods and the low number of participants. Longer follow‐up periods would help to record whether change is sustained but may increase the number of dropouts due to constantly changing health care and frequent staff turnover (Dichter et al. 2017). The low number of participants, in turn, reduces the generalisability of the effects of the intervention (Moore et al. 2021). The methodological weaknesses of intervention studies require attention to be paid to their more careful planning in the future. Despite the quality shortcomings, these studies remain valuable, providing insights for developing the potential components of WAW interventions.

Some of the included studies were quite old (i.e., Berg, Welander Hansson, and Hallberg 1994), and as health systems have changed radically over time, the interventions as such may no longer be exploitable. Existing WAW interventions were targeted to the entire care staff. Although qualified nurses may not always be the primary group in care settings for older people, they play an essential role in providing more diverse nursing care for older people (OECD 2021). Thus, specific aspects of the work carried out by qualified and non‐qualified health professionals in these settings can be better addressed in WAW interventions.

Interventions were typically complex, including more than one component. Achieving straightforward results showing the causal relationship is challenging due to the often complex nature of the interventions, with several interconnected components or effect mechanisms. In addition, work environments and systems are usually complex in health care, which may lead to challenges in verifying the effectiveness of interventions on WAW (Moore et al. 2021).

Interventions developed to improve nurses' WAW focused on nurses' health, nursing care, care facilities and management. Mostly, the interventions focused on nurses' physical or mental health. Those interventions which had positive effect on physical outcomes included consultation or computer‐based training as one component. Likewise, one intervention that included gamified training as a component had resulted in positive psychosocial outcomes. However, more research is needed of these active learning strategies on improving nurses' WAW as these were from individual studies. In the previous reviews, the most frequent health aim of interventions has been mental health (Melnyk et al. 2020; Schaller et al. 2022) also in care settings for older people (Nikunlaakso et al. 2022). Concentration on mental health interventions is evident as mental health issues are described in the literature as common among nurses (NASEM 2021).

The second most common intervention focused on nursing care followed by interventions focusing on care facilities. Mostly, those interventions that had positive effects on physical, psychosocial and environmental outcomes included components of training and active exercises on nursing care or with care equipment. At best, these kinds of interventions potentially support both the nurses' work well‐being and the quality of care for older people (Asuquo, Tighe, and Bradshaw 2021). However, more interventions focusing on care facilities are warranted as there is a growing shortage of nurses and a decrease in 24‐h units for older people (OECD 2020). With our search strategy, we discovered only one intervention that focused on management although leadership is acknowledged in the literature as one of the most important facilitators of nurses' WAW. (Schwendimann et al. 2016; Lee et al. 2020). Further review of the literature on management focus interventions that aim to improve nurses' WAW is needed to identify the need for these kinds of interventions.

According to the results of this review, there were statistically significant positive outcomes reached with interventions in both quasi‐experimental and RCT designs. Interventions resulted in a range of outcomes in the physical, psychosocial or environmental dimensions of WAW. However, interventions that focus on the ethical dimension of WAW were not discovered. This dimension needs to be considered in future research as nurses confront ethical challenges and constraints in their daily practice that affect negatively on their moral well‐being (NASEM 2021). Nearly all the interventions showed some positive outcomes, albeit with instances of negative and neutral results. One moderate‐quality quasi‐experimental study (Nelson et al. 2006) and one high‐quality RCT (Tveito and Eriksen 2009) with interventions focusing on nurses' health showed positive outcomes on all the dimensions of WAW. Interventions that focused on nurses' health, nursing care and nursing facilities all showed positive outcomes despite the study design or quality of the study. One moderate‐quality quasi‐experimental study (Dichter et al. 2017) and one moderate‐quality RCT (Zimmerman et al. 2010) with interventions focusing on nursing care showed negative outcomes on the psychosocial and environmental dimensions of WAW. Interventions focusing on nurses' health or care facilities can be promising to improve nurses' WAW. This is supported by previous literature (Asuquo, Tighe, and Bradshaw 2021). Management‐focused intervention had positive outcomes only on the psychosocial dimension of WAW; however, there was only one high‐quality RCT study focusing on management (Jeon et al. 2015). Therefore, the role of management in improving nurses' WAW would need further examination.

### 4.1 Limitations and Strengths

This review has both limitations and strengths. As the first limitation, the literature search was conducted by one researcher. The second limitation is that we included only studies written in English, leading to a potential selection bias. As a third limitation, we included all studies irrespective of their quality, which carries implications for the generalisation of the results and their future utilisation. Finally, we included studies involving qualified nurses and other health care professionals which potentially limit the review findings' applicability to nurses in particular.

The strengths of this review include, firstly, the adherence to a registered review protocol. Secondly, we employed a comprehensive search strategy, crafting search terms to encompass various spellings of WAW and meeting the specific requirements of each database and platform. We conducted searches, without time limitations, and complemented these searches with manual backward citation chasing to identify all relevant literature. To maintain accuracy, we employed Zotero reference management software, to handle search records and screening. Thirdly, two researchers (JW and SK) conducted the screening process initially working independently to prevent biased selection and then collaboratively to reach a consensus on the included articles. Fourthly, we followed the initial analysis of one researcher (JW) and scrutinised and validated the results by the research group, ensuring rigour and reliability.

### 4.2 Implications for Practice, Stakeholders and Researchers

According to the findings of this study, we present implications for practice, stakeholders (policymakers, health care managers and nurse educators) and researchers. Strategies and interventions aimed at improving nurses' WAW are needed in practice as nurses' WAW is crucial in recruiting to and retaining nurses in care settings for older people. Moreover, promoting WAW also contributes to the quality of care for older people and the provision of ethically high‐quality health services. Policymakers and health care managers at various levels can address the improvement of WAW of nurses working in care settings for older people, considering various facets that span from nurses' health and sustainable care environment with a healthy climate to patient care and management practices. Nurse educators can more systematically integrate WAW into teaching to cultivate a positive outlook on work among students, emphasising ways to support their well‐being in the workplace including care settings for older people. For researchers, the active involvement of nurses themselves and WAW support representatives (such as nurse managers, occupational health professionals, nurse educators and policymakers) is crucial in the development and implementation of interventions to maximise the likelihood of positive health impacts and sustained changes also for future generations.

## 5 Conclusion

Interventions to improve the WAW of nurses working in care settings for older people have focused on various aspects, namely nurses' health, nursing care, care facilities and management. Interventions have resulted in various outcomes, and those focusing on nurses' health or care facilities can be promising to improve nurses' WAW. Interventions focusing on nursing care can also have negative outcomes for nurses' WAW. Further meticulously planned intervention studies are needed for sustainable and long‐term effects on WAW of nurses working in care settings for older people.

## Author Contributions

Substantial contributions to the conception or design of the work, or the acquisition, analysis or interpretation of data for the work: J.W., A.S., H.L.‐K., M.S., R.S. and S.K. Drafting the work or reviewing it critically for important intellectual content: J.W., A.S., H.L.‐K., M.S., R.S. and S.K. Final approval of the version to be published: J.W., A.S., H.L.‐K., M.S., R.S. and S.K. Agreement to be accountable for all aspects of the work in ensuring that questions related to the accuracy or integrity of any part of the work are appropriately investigated and resolved: J.W., A.S., H.L.‐K., M.S., R.S. and S.K.

## Conflicts of Interest

The authors declare no conflicts of interest.

## Supporting information


Appendix S1

Appendix S2

## Data Availability

Data sharing is not applicable to this article as no new data were created or analyzed in this study.
