# Supplementary material for: Interventions Intended to Improve the Well‐Being at Work of Nurses Working in Care Settings for Older People—A Systematic Review
Source: Int J Older People Nurs. 2024 Dec 25;20(1):e70005. doi: 10.1111/opn.70005 (PMC11669382; doi:10.1111/opn.70005)
Supplement: Supplementary file 1 — Appendix S1 [file OPN-20-e70005-s002.docx]

Appendix S1. Search strategy

| **Database** | **Search terms** |
| --- | --- |
| PubMed | (nurs*[tw]) AND ("well-being" OR "well being" OR wellbeing* OR "occupational health") AND (work*[tw] OR job[tw] OR occupational*[tw] OR employee*[tw]) AND ("aged service*"[tw] OR "aged care" OR "elderly service*" OR "elderly care" OR "older people's service*" OR "older people service*" OR "services for older people" OR "older people care" OR "older people nursing" OR "long-term care" OR "long-term service*" OR "nursing home*" OR "care home*" OR "home care service*" OR "home care" OR "homecare" OR "residential facilit*" OR "residential care" OR "sheltered housing" OR "supported living" OR "assisted living" OR "geriatric care" OR "geriatric nursing"[tw] OR "gerontological nursing"[tw] OR "gerontological care"[tw] OR "gerontologic nursing"[tw] OR geriatric*[tw] OR gerontolog*[tw]) |
| CINAHL | (nurs*) AND ("well-being" OR "well being" OR wellbeing* OR "occupational health") AND (work* OR job OR occupational* OR employee*) AND ("aged service*" OR "aged care" OR "elderly service*" OR "elderly care" OR "older people's service*" OR "older people service*" OR "services for older people" OR "older people care" OR "older people nursing" OR "long-term care" OR "long-term service*" OR "nursing home*" OR "care home*" OR "home care service*" OR "home care" OR "homecare" OR "residential facilit*" OR "residential care" OR "sheltered housing" OR "supported living" OR "assisted living" OR "geriatric care" OR "geriatric nursing" OR "gerontological nursing" OR "gerontological care" OR "gerontologic nursing" OR geriatric* OR gerontolog*) |
| APA PsycINFO | (nurs*) AND ("well-being" OR "well being" OR wellbeing* OR "occupational health") AND (work* OR job OR occupational* OR employee*) AND ("aged service*" OR "aged care" OR "elderly service*" OR "elderly care" OR "older people's service*" OR "older people service*" OR "services for older people" OR "older people care" OR "older people nursing" OR "long-term care" OR "long-term service*" OR "nursing home*" OR "care home*" OR "home care service*" OR "home care" OR "homecare" OR "residential facilit*" OR "residential care" OR "sheltered housing" OR "supported living" OR "assisted living" OR "geriatric care" OR "geriatric nursing" OR "gerontological nursing" OR "gerontological care" OR "gerontologic nursing" OR geriatric* OR gerontolog*) |
| Cochrane Library  Title Abstract Keyword | (nurs*) AND (well-being OR well being OR wellbeing* OR occupational NEXT health) AND (work* OR job OR occupational* OR employee*) AND (aged NEXT service* OR aged NEXT care OR elderly NEXT service* OR elderly NEXT care OR older NEXT people's NEXT service* OR older NEXT people NEXT service* OR services NEXT older NEXT people OR older NEXT people NEXT care OR older NEXT people NEXT nursing OR long-term NEXT care OR long-term NEXT service* OR nursing NEXT home* OR care NEXT home* OR home NEXT care NEXT service* OR home NEXT care OR homecare OR residential NEXT facilit* OR residential NEXT care OR sheltered NEXT housing OR supported NEXT living OR assisted NEXT living OR geriatric NEXT care OR geriatric NEXT nursing OR gerontological NEXT nursing OR gerontological NEXT care OR gerontologic NEXT nursing OR geriatric* OR gerontolog*) |
| Web of Science  Topic | (nurs*) AND ("well-being" OR "well being" OR wellbeing* OR "occupational health") AND (work* OR job OR occupational* OR employee*) AND ("aged service*" OR "aged care" OR "elderly service*" OR "elderly care" OR "older people's service*" OR "older people service*" OR "services for older people" OR "older people care" OR "older people nursing" OR "long-term care" OR "long-term service*" OR "nursing home*" OR "care home*" OR "home care service*" OR "home care" OR "homecare" OR "residential facilit*" OR "residential care" OR "sheltered housing" OR "supported living" OR "assisted living" OR "geriatric care" OR "geriatric nursing" OR "gerontological nursing" OR "gerontological care" OR "gerontologic nursing" OR geriatric* OR gerontolog*) |
| Scopus | TITLE-ABS-KEY ((nurs*) AND ("well-being" OR "well being" OR wellbeing* OR "occupational health") AND (work* OR job OR occupational* OR employee*) AND ("aged service*" OR "aged care" OR "elderly service*" OR "elderly care" OR "older people's service*" OR "older people service*" OR "services for older people" OR "older people care" OR "older people nursing" OR "long-term care" OR "long-term service*" OR "nursing home*" OR "care home*" OR "home care service*" OR "home care" OR "homecare" OR "residential facilit*" OR "residential care" OR "sheltered housing" OR "supported living" OR "assisted living" OR "geriatric care" OR "geriatric nursing" OR "gerontological nursing" OR "gerontological care" OR "gerontologic nursing" OR geriatric* OR gerontolog*)) |
