# Supplementary material for: Interventions Intended to Improve the Well‐Being at Work of Nurses Working in Care Settings for Older People—A Systematic Review
Source: Int J Older People Nurs. 2024 Dec 25;20(1):e70005. doi: 10.1111/opn.70005 (PMC11669382; doi:10.1111/opn.70005)
Supplement: Supplementary file 2 — Appendix S2 [file OPN-20-e70005-s001.docx]

Appendix S2. Quality appraisal for studies according to Joanna Briggs Institute checklists for quasi-experimental studies and randomized controlled trials.

| **Publication/**  **JBI checklist for quasi-experimental studies** | **Ericson-Lidman & Åhlin 2017** | **Johnsson et al. 2002** | **Nelson et al. 2006** | **Petterson et al. 2006** | **Sarabia-Cobo et al. 2017** | **Dichter et al., 2017** | **Engst et al., 2005** | **Engst et al., 2004** | **Miller et al., 2006** | **Zwakhalen et al., 2018** |  |
| --- | --- | --- | --- | --- | --- | --- | --- | --- | --- | --- | --- |
| 1. Is it clear in the study what is the ‘cause’ and what is the ‘effect’ (i.e. there is no confusion about which variable comes first)? | Yes | Yes | Yes | Yes | Yes | Yes | Yes | Unclear | Yes | Yes |  |
| 2. Were the participants included in any comparisons similar? | Yes | Yes | No | Yes | Yes | No | Unclear | Yes | No | No |  |
| 3. Were the participants included in any comparisons receiving similar treatment/care, other than the exposure or intervention of interest? | Yes | Yes | Yes | Unclear | Yes | Unclear | Yes | Yes | Unclear | Unclear |  |
| 4. Was there a control group? | No | Yes | No | No | No | Yes | Yes | Yes | Yes | Yes |  |
| 5. Were there multiple measurements of the outcome both pre and post the intervention/ exposure? | Yes | Yes | Yes | Yes | Yes | Yes | Yes | Yes | Yes | Yes |  |
| 6. Was follow up complete and if not, were differences between groups in terms of their follow up adequately described and analyzed? | No | Yes | Unclear | Yes | Yes | Yes | Yes | Unclear | Unclear | Yes |  |
| 7. Were the outcomes of participants included in any comparisons measured in the same way? | Yes | Yes | Yes | Yes | Yes | Yes | Yes | Yes | Yes | Yes |  |
| 8. Were outcomes measured in a reliable way? | Yes | Yes | Unclear | Yes | Yes | Yes | Yes | Unclear | Yes | Yes |  |
| 9. Was appropriate statistical analysis used? | No | Unclear | Unclear | Unclear | Unclear | Unclear | No | Unclear | Unclear | Unclear |  |
| **Publication/**  **JBI checklist for randomized controlled trials** | **Berendonk et al., 2019** | **Berg et al., 1994** | **Buruck et al., 2016** | **Davison et al. 2007** | **Faghri et al. 2017** | **Hurtado et al. 2016** | **Jeon et al. 2015** | **Kloos et al. 2019** | **Torsney et al. 2011** | **Tveito & Eriksen, 2009** | **Zimmerman et al., 2010** |
| 1. Was true randomization used for assignment of participants to treatment groups? | Yes | No | No | Unclear | No | Unclear | Yes | Unclear | Yes | Yes | Yes |
| 2. Was allocation to treatment groups concealed? | Yes | No | No | Unclear | Unclear | Yes | Yes | No | Unclear | Yes | Yes |
| 3. Were treatment groups similar at the baseline? | Yes | Unclear | Yes | No | Yes | Yes | Yes | No | Unclear | Yes | Yes |
| 4. Were participants blind to treatment assignment? | Unclear | Unclear | No | Unclear | Unclear | Unclear | Yes | No | No | No | No |
| 5. Were those delivering treatment blind to treatment assignment? | No | Unclear | No | No | Unclear | No | No | No | No | No | No |
| 6. Were outcomes assessors blind to treatment assignment? | Unclear | Unclear | Unclear | Unclear | Unclear | Unclear | Yes | No | No | Yes | No |
| 7. Were treatment groups treated identically other than the intervention of interest? | Unclear | Yes | Unclear | Yes | Yes | Unclear | Yes | Yes | No | Yes | Yes |
| 8. Was follow up complete and if not, were differences between groups in terms of their follow up adequately described and analyzed? | No | No | No | No | Yes | Yes | Yes | Yes | Yes | Yes | Yes |
| 9. Were participants analyzed in the groups to which they were randomized? | Yes | Yes | Not applicable | Yes | Yes | Yes | Yes | Yes | Unclear | Yes | Yes |
| 10. Were outcomes measured in the same way for treatment groups? | Yes | Yes | Yes | Yes | Yes | Unclear | Yes | Yes | Yes | Yes | Yes |
| 11. Were outcomes measured in a reliable way? | Unclear | No | Unclear | No | Unclear | No | Yes | Unclear | Yes | Yes | Yes |
| 12. Was appropriate statistical analysis used? | No | No | Unclear | No | No | Yes | Yes | Yes | No | Yes | Yes |
| 13. Was the trial design appropriate, and any deviations from the standard RCT design (individual randomization, parallel groups) accounted for in the conduct and analysis of the trial? | Yes | No | Yes | Yes | Yes | Yes | Yes | Yes | Unclear | Yes | Yes |
